# Asymmetrical middle cerebral artery bifurcations are more vulnerable to aneurysm formation

**DOI:** 10.1038/s41598-019-51734-4

**Published:** 2019-10-24

**Authors:** Xue-Jing Zhang, Wei-Li Hao, Dong-Hai Zhang, Bu-Lang Gao

**Affiliations:** 1 Department of Medical Research, Shijiazhuang First Hospital, Hebei Medical University, 36 Fanxi Road, Shijiazhuang, Hebei Province 050011 China; 2 Henan Balance Medical Laboratory, Henan Balance Medical Corporation Ltd, Zhengzhou, Henan Province 450000 China

**Keywords:** Predictive markers, Stroke

## Abstract

The objective of this study was to elucidate possible relationship between middle cerebral artery (MCA) bifurcation aneurysms and bifurcation morphology. In the present study, 799 patients with three-dimensional angiography were enrolled, including 115 patients with MCA bifurcation aneurysms and 684 subjects without aneurysms. The MCA bifurcation geometry, including angles formed between two M2 segments (φ1) and between M1 and M2 segments, vessel diameters and aneurysm sizes were measured. DA ratio (larger/smaller M2 in diameter) and LA ratio (larger/smaller lateral angle) were also analyzed. The LA and DA ratios and angle φ1 were significantly (*P* < 0.0001) greater in patients harboring MCA bifurcation aneurysms than in the control, whereas lateral angles and bifurcation branch diameters were significantly smaller (*P* < 0.01) in patients with than without bifurcation aneurysms. Angle φ1 was significantly increased (*P* < 0.0001) while both lateral angles significantly decreased (*P* < 0.0001 and *P* = 0.0005, respectively) with increase of patients’ age. The size of MCA bifurcation aneurysms was significantly (*P* < 0.05) positively correlated with the bifurcation vascular diameter and aneurysm neck at the MCA bifurcation. A significantly positive correlation existed between aneurysm neck and DA ratio (*P* = 0.0075), whereas an inverse correlation between aneurysm neck and LA ratio (*P* = 0.0219). MCA bifurcation aneurysms were mostly deviated toward the smaller lateral angles and smaller M2 branch. In conclusion, aneurysmal MCA bifurcations have asymmetrical bifurcation structures with widened bifurcation angles, narrowed lateral angles, decreased M1 diameter, imbalanced lateral angles and M2 segments, with the cutoff bifurcation angle of 125.0° and cutoff lateral angle ratio of 1.57 for predicting MCA bifurcation aneurysms, whereas normal MCA bifurcations show close to symmetrical structures in the lateral angles and M2 branches.

## Introduction

Cerebral aneurysms are a primary health issue affecting approximately 2–4% of the general population^1,2^. The middle cerebral artery (MCA) bifurcation is a preferred site for aneurysm formation. It is revealed that MCA bifurcation aneurysms account for 18–36% of all cerebral aneurysms^3^, with an annual rupture rate of 0.36%^4^. MCA aneurysms were difficult to be treated endovascularly because of complex branching patterns of the parent vessels^5^. Because of the complex morphology, high prevalence and rupture rate of MCA aneurysms, elucidation of local bifurcation morphological patterns more vulnerable to aneurysm formation may be beneficial to risk assessment of aneurysm initiation.

It has been hypothesized that hemodynamic stresses are the initial factor for aneurysm formation, and cerebral aneurysms are common at arterial bifurcations and junctions where vessel walls are exposed to abruptly increased hemodynamic stresses^6^. Alteration of arterial bifurcation anatomy, including bifurcation angle and branch diameters, is associated with diversification of hemodynamic stresses at the arterial bifurcation apex^7^. After analyzing the MCA bifurcation morphology in 62 patients, Sadatomo *et al*.^2^ demonstrated that normal MCA bifurcations were close to a symmetric structure, whereas aneurysmal bifurcations did not show this symmetry. However, the correlation of aneurysm presence with bifurcation morphology, including bifurcation and lateral angles and branch diameter, was not thoroughly examined in this study.

Several studies have revealed that wider bifurcations, including the anterior cerebral artery (ACA)^8^, basilar artery (BA)^9^ and MCA bifurcations^7^, are associated with aneurysm presence, inferring that bifurcation angle widening can increase hemodynamic stresses to promote aneurysm formation. Furthermore, our previous studies showed that most ACA and BA bifurcation aneurysms occurred on bifurcations with widened bifurcation angles and were deviated to the smaller lateral angle and smaller branch vessel^8,9^, and few studies have been performed on the association of aneurysm presence with MCA bifurcation geometry, especially the lateral angels and bilateral branch correlation with MCA aneurysm presence. We hypothesized that MCA bifurcation aneurysms also occurred on asymmetrical MCA bifurcations with wider bifurcation angle and deviated to the smaller lateral angle and smaller branch vessel. In the current study, we consequently sought to evaluate the anatomy differences between the bifurcations with and without MCA aneurysms for a possible link in the MCA bifurcation aneurysm presence with MCA bifurcation geometry.

## Methods

### Patient selection

Between March 2004 and February 2015, consecutive patients who had undergone three-dimensional digital subtraction angiography (DSA) in our hospital were enrolled in this study. All data with sufficient imaging quality to permit accurate morphologic analysis were enrolled, and 18 patients with poor three-dimensional angiography were excluded. A total of 115 patients with MCA aneurysms were eligible for analysis, including 32 patients with subarachnoid hemorrhage (SAH), 26 with headache, 19 with cerebrovascular disease and 38 which were incidentally found to have MCA aneurysms. Nonaneurysmal MCA bifurcations were evaluated in 684 patients who had digital subtraction angiography for suspected cerebrovascular diseases. Data on patients’ age, gender, aneurysm location, clinical risk factors and symptoms were collected from a prospectively maintained database. This study was approved by the ethics committee of Shijiazhuang First Hospital, Hebei Medical University, with all patients given the signed informed consent. All methods were performed in accordance with the relevant guidelines and regulations.

### Morphological features analysis

**Figure 1:**
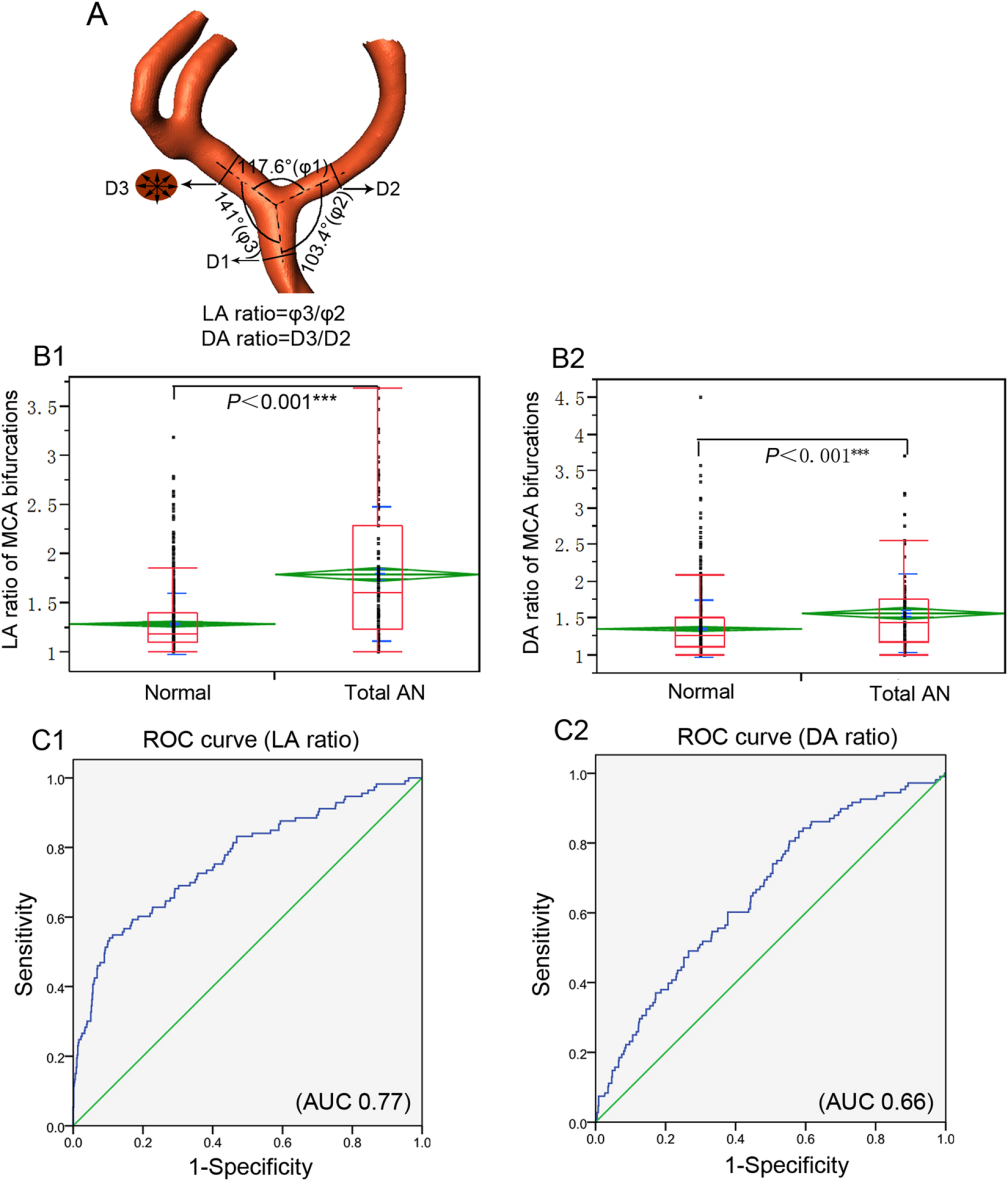
(**A**) Schematic drawing shows measurement of bifurcation angles and vessel diameters. φ1, the angle formed between two M2 segments; φ2, the smaller angle formed between M1 and one M2 segment of middle cerebral artery (MCA); φ3, the larger angle between M1 and the other M2 segment of MCA; D1, the diameter of M1; D2, diameter of the smaller M2 branch; D3, diameter of the larger M2 branch. LA ratio was defined as φ3/φ2 and DA ratio was named as D3/D2. (**B**) LA and DA ratios of MCA bifurcations between normal and aneurysmal cases. Normal, control subjects without MCA aneurysms; total AN, MCA bifurcations harboring aneurysms. (C1,C2) Adjusted ROC curves of LA and DA ratios for predicting aneurysm presence. AUC, area under the curve. Differences were considered statistically significant at *P* < 0.05.

Three-dimensional rotational angiography data were reconstructed and transferred for surface rendering by using Amira software (version 5.2.2, Visage Imaging, San Diego, CA, USA). The angles formed between M1 and lateral M2 branches (angle φ2 and φ3, respectively) and between two M2 segments (angle φ1) were measured as stated in a previous study^9^. Briefly, the φ1 angle was measured by use of 3 dots after the central point was placed at the tip of the bifurcation in line with the central axis of the M1 segment of MCA, and the other 2 dots marked the central axis of the lateral M2 segment. The angles φ2 and φ3 were evaluated in a similar manner (Fig. 1A). The smaller lateral angle between M1 and M2 was termed angle φ2, and the larger one between M1 and M2 was called angle φ3. Larger lateral angle/smaller lateral angle was termed as the LA ratio.

The diameter of the M1 segment of MCA was measured midway between the internal carotid artery bifurcation and the main branching point of MCA, similar to the method provided by Ingebrigtsen *et al*.^10^. Both M2 diameters were measured 5 mm beyond the MCA bifurcation apex. The diameter was measured on four different centripetal axes in a closer to circular cross-section that oriented 90° to the flow axis (Fig. 1A). The M1 diameter was termed D1, and the diameter of smaller and larger M2 branches was named D2 and D3, respectively. The diameter of larger M2/diameter of smaller M2 was defined as the DA ratio. The maximum height (maximal distance from the center of the aneurysm neck to the highest point on the aneurysm dome), maximum width (maximal transverse diameter) and aneurysm neck (maximal diameter at the neck) were measured similar to the approach used by previous reports^11,12^.

**Figure 2:**
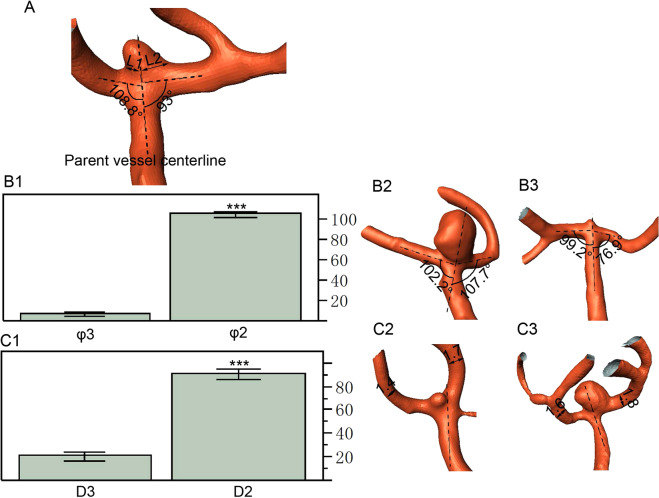
(**A**) Schematic drawing shows measurement of aneurysm deviation. Bidirectional arrows represent the length of the aneurysm neck (L1 and L2) divided by the central line of parent artery M1. (**B**) Most of the middle cerebral artery (MCA) bifurcation aneurysms deviated towards the smaller angle φ2 formed between M1 segment and one M2 segment. (B2,B3) The MCA aneurysm deviated to the smaller lateral angle 102.2° (B2) but to the larger lateral angle 99.2° (B3). (**C**) Most of the MCA bifurcation aneurysms deviated towards the smaller artery branch (D2). (C2,C3) The aneurysm deviated to the smaller diameter (1.4 mm) branch (C2) but towards the larger diameter (1.8 mm) branch (C3). ^***^P < 0.001.

Aneurysm deviation was also measured (Fig. 2A) similar to our previous report^8^. Briefly, according to the intersection of aneurysm neck with the longitudinal axis of parent artery, the length of aneurysm neck was divided into two sections, defined as L1 and L2. The MCA aneurysm deviation was decided based on the length of the two sections of L1 and L2. In L1 > L2, the aneurysm deviated towards the L1 side while in L1 < L2, the aneurysm deviated towards the L2 side.

### Statistical analysis

The JMP statistical software (version 10.01.2, SAS Institute, Cary, NC, USA) was used for statistical analysis. The *t*-test and the chi-square analysis were used to analyze age and enumeration data between two groups. Covariance analysis was used to examine the morphology parameter differences between aneurysmal and normal groups, including covariates of age, family history and blood glucose concentration. Adjusted receive operator characteristic (ROC) analysis was performed to determine the area under the curve (AUC), as well as optimal cutoff values for LA ratio, DA ratio, bifurcation angles and vessel diameters between aneurysmal and nonaneurysmal MCA bifurcations. The morphologic feature correlation was assessed by multivariate analysis using least squares linear regression. *P* value < 0.05 was considered to be statistically significant.

## Results

Among 799 patients enrolled, 115 patients had MCA bifurcation aneurysms while 684 control patients had no aneurysms. There was no significant (*P* > 0.05) difference in the demographic (age, sex) and clinical risk factors (hypertension, smocking, diabetes and family history) between patients with and without aneurysms (Table 1).

**Table 1:** Baseline characteristics of patients with and without MCA bifurcation aneurysms.

	Normal (n = 684)	With AN (n = 115)	*P*-value
Gender			
Female	481	84	0.55
Male	203	31	
Mean age in yrs	54.7 ± 13.9	58.8 ± 13.5	0.06
Hypertension			
Yes	229	46	0.17
No	455	69	
Smocking history			
Yes	312	61	0.14
No	372	54	
Diabetes			
Yes	202	43	0.09
No	482	72	
Family history			
Yes	68	18	0.07
No	616	97	

Note: MCA, middle cerebral artery; AN, aneurysm.

**Figure 3:**
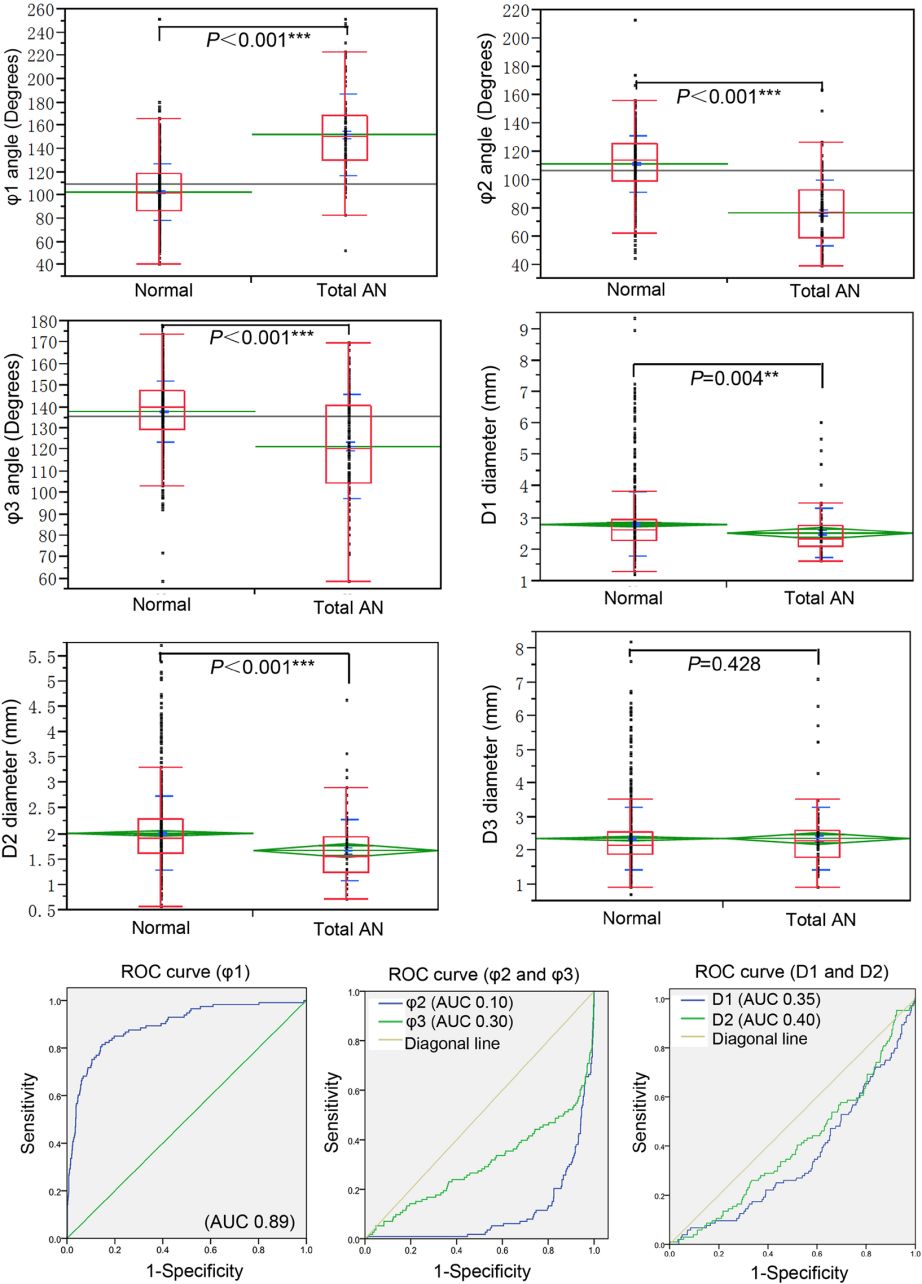
Box plots display significant differences in the mean value of angles (φ1, φ2 and φ3), vessel diameters (D1, D2 and D3), and adjusted ROC curves analysis of bifurcation angle (φ1, φ2 and φ3) and vessel diameter (D1 and D2) for predicting aneurysm presence. Total AN, patients with middle cerebral artery (MCA) bifurcation aneurysms; Normal, control subjects with no aneurysms; φ1, the angle formed between two M2 segments; φ2 and φ3 indicate the smaller and larger angles formed between M1 and one M2 segment of MCA, respectively. D1, the diameter of M1; D2 and D3, the diameter of the smaller and larger M2 segments, respectively. AUC, area under the curve. Differences were considered statistically significant at *P* < 0.05.

LA and DA ratio of normal MCA were 1.3 ± 0.3 (0.5–3.2) and 1.4 ± 0.4 (0.9–4.5), respectively, which were statistically smaller than aneurysmal cases (1.8 ± 0.7 and 1.6 ± 0.5, respectively) (*P* < 0.001) (Table 2 and Fig. 1). Both φ2 and φ3 were statistically greater in normal (111.3° ± 19.3° and 138.2° ± 14.8°, respectively) than aneurysmal (76.6° ± 22.9° and 122.2° ± 24.1°, respectively) MCA bifurcations (*P* < 0.001), however, φ1 was significantly smaller in normal (102.8° ± 24.7°) than aneurysmal (152.4° ± 35.0°) MCA bifurcations (*P* < 0.001) (Table 2 and Fig. 3). The D1 and D2 values were significantly larger in normal (2.8 ± 1.0 and 1.9 ± 0.7, respectively) than aneurysmal (2.5 ± 0.8 and 1.6 ± 0.6, respectively) MCA bifurcations (*P* < 0.01 and *P* < 0.001, respectively) (Table 2 and Fig. 3).

**Table 2:** Bifurcation angles, branch diameters, LA and DA ratios of MCA bifurcation.

	Normal	Total AN
φ1 (°)	102.8 ± 24.7 (40–251.4)	152.4 ± 35.0^***^ (51.4–251.4)
φ2 (°)	111.3 ± 19.3 (43.7–173.8)	76.6 ± 22.9^***^ (38.7–163.3)
φ3 (°)	138.2 ± 14.8 (58.4–212.7)	122.2 ± 24.1^***^ (58.8–169.5)
D1(mm)	2.8 ± 1.0 (1.2–9.3)	2.5 ± 0.8^**^ (1.6–6.0)
D2 (mm)	1.9 ± 0.7 (0.6–5.2)	1.6 ± 0.6^***^ (0.7–4.6)
D3 (mm)	2.5 ± 0.9 (1.0–8.2)	2.4 ± 0.9 (1.3–7.1)
LA ratio	1.3 ± 0.3 (0.5–3.2)	1.8 ± 0.7^***^ (0.4–3.7)
DA ratio	1.4 ± 0.4 (0.9–4.5)	1.6 ± 0.5^***^ (0.8–3.7)

Note: Data are shown as mean ± standard deviation. MCA, middle cerebral artery; AN, aneurysm; D2, smaller M2 diameter; D3, larger M2 diameter; φ 1, MCA bifurcation angle between two M2 segments; φ2, the smaller lateral angle formed between M1 and one M2 segment; φ3, the larger lateral angle formed between M1 and the other M2 segment. LA ratio and DA ratio were defined as φ3/φ2 and D3/D2, respectively. ^**^P < 0.01 and ^***^P < 0.001 compared with normal MCA bifurcations.

The ROC curve was used to differentiate morphological features of normal from aneurysmal cases. The optimal threshold value of LA ratio distinguishing between normal and aneurysmal MCA bifurcations was 1.57 (AUC, 0.77), with 54.9% sensitivity and 88.7% specificity (Fig. 1C1). The AUC of DA ratio (Fig. 1C2), φ2, φ3, D1 and D2 (Fig. 3) were 0.66, 0.10, 0.30, 0.35 and 0.40, respectively. The bifurcation angle φ1 was the best performer in discriminating between aneurysmal and nonaneurysmal MCA bifurcations, with the optimal φ1 threshold of 125.0° (AUC, 0.89) and the sensitivity and specificity of 81.4% and 85.4%, respectively (Fig. 3).

**Figure 4:**
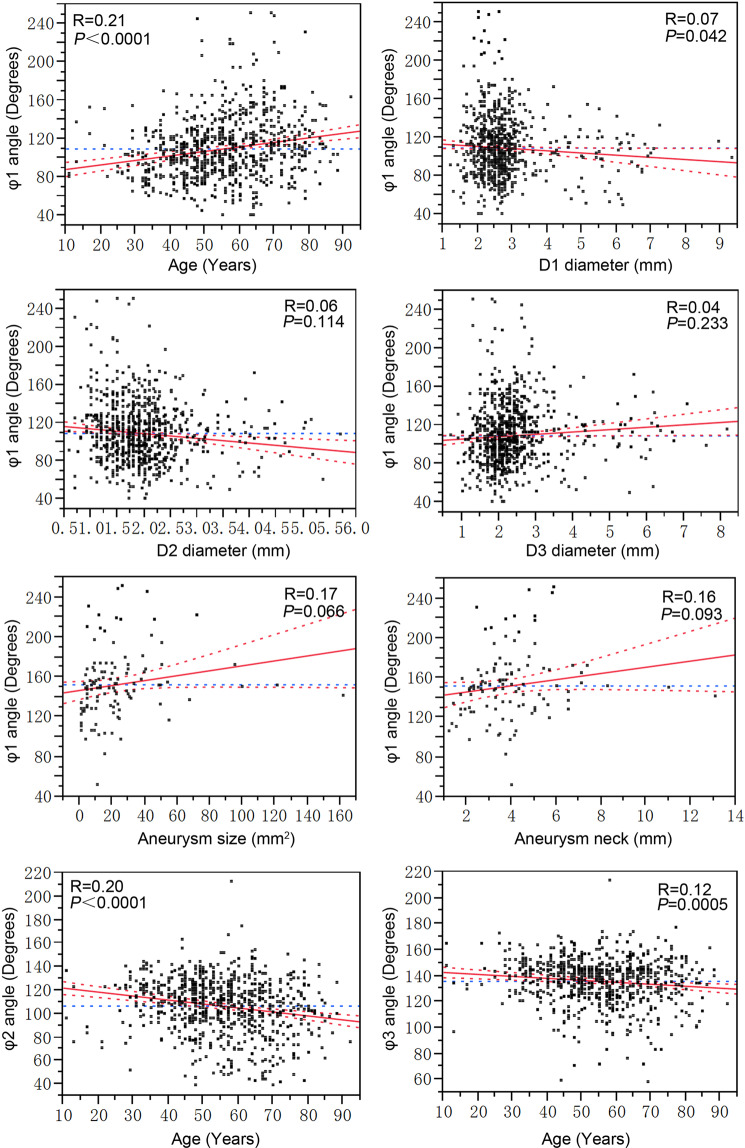
Multiple regression analysis shows correlation of angle φ1 with age and vessel diameter (D1, D2 and D3) in all patients or with aneurysm size in patients with middle cerebral artery (MCA) bifurcation aneurysms, as well as correlation of angles φ2 and φ3 with age. Aneurysm size was represented by aneurysm maximum width × maximum height. φ1, the angle formed between two M2 segments; φ2, the smaller angle formed between M1 and one M2 segment of MCA; φ3, the larger angle between M1 and the other M2 segment of MCA; D1, D2 and D3, diameter of the M1, smaller M2 and larger M2 segments, respectively. Differences were considered statistically significant at *P* < 0.05.

Given the important role of φ1 in aneurysm formation prediction, multivariate analysis employing the least squares linear regression was used to investigate the relative dependency of angle φ1 on age, MCA bifurcation and aneurysm geometry. A statistically significant (R = 0.21, *P* < 0.0001) positive linear correlation existed between angle φ1 and age, and an inverse correlation in angles φ2 or φ3 with age (*P* < 0.0001 and *P* = 0.0005, respectively) in all patients. A significant inverse correlation was observed between angle φ1 and diameter D1 (R = 0.07, *P* = 0.042). No significant correlations were present between angle φ1 and D2 (*P* = 0.114), D3 (*P* = 0.233), aneurysm size (maximal height × maximal width) (*P* = 0.066) or aneurysm neck (*P* = 0.093) in patients harboring MCA aneurysms (Fig. 4).

**Figure 5:**
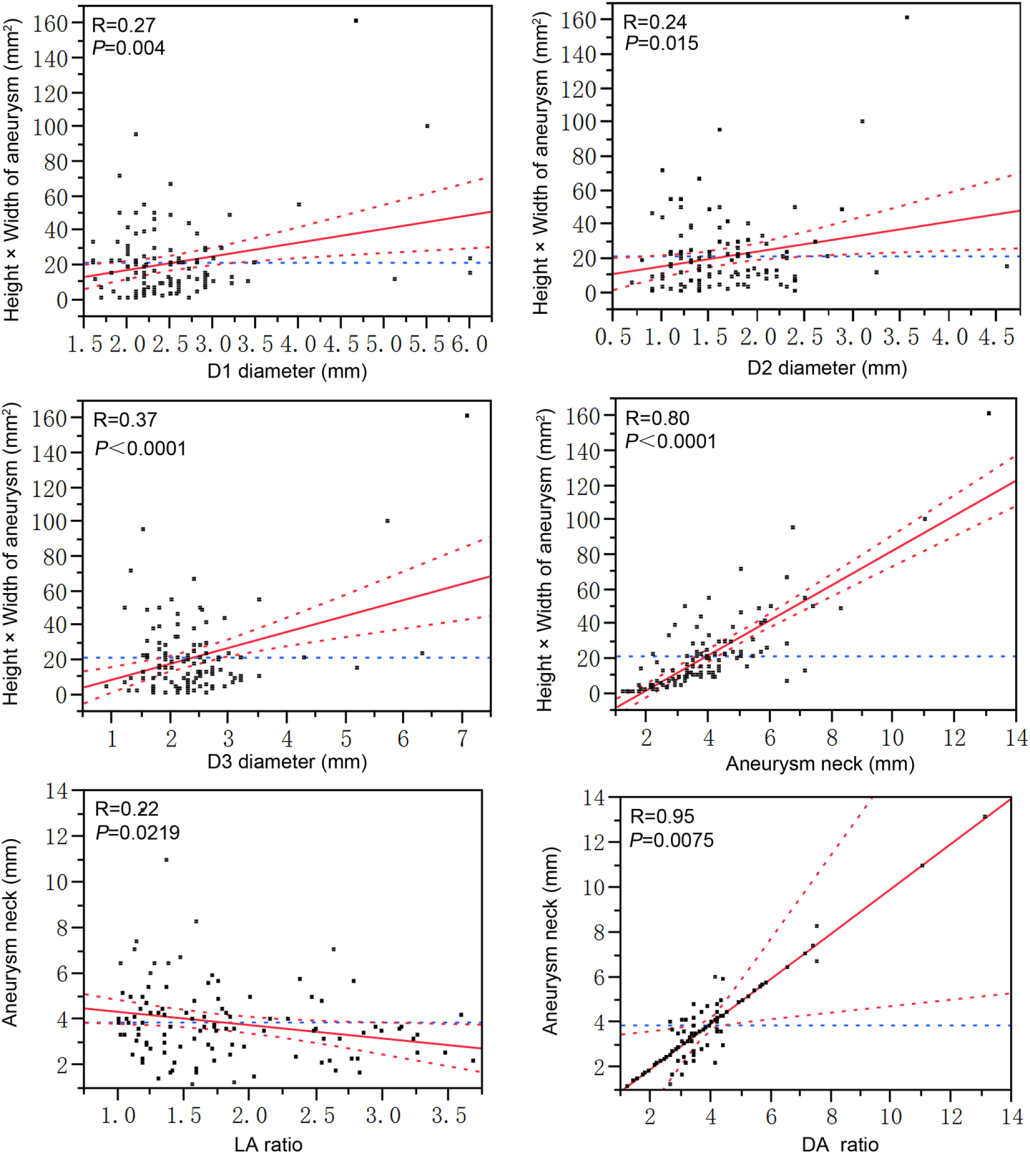
Multiple regression analysis shows the correlation of middle cerebral artery (MCA) aneurysm size with the vessel diameter, aneurysm neck, LA and DA ratio in patients with MCA bifurcation aneurysms. Aneurysm size is represented by aneurysm maximum height × width. D1, the diameter of M1; D2, the diameter of the smaller M2; D3, diameter of the larger M2; LA ratio, larger lateral angle (φ3)/smaller lateral angle (φ2); DA ratio, D3/D2. Differences were considered statistically significant at *P* < 0.05.

In 115 patients harboring MCA bifurcation aneurysms, a significant positive linear correlation was also observed between aneurysm size and bifurcation branch diameters (D1, D2 and D3) (*P* = 0.004, *P* = 0.015 and *P* < 0.0001, respectively) or aneurysm neck (R = 0.80, *P* < 0.0001) (Fig. 5). No significant (*P* > 0.05) correlations existed between branch diameter and patients’ age. Furthermore, linear regression analysis showed a significant positive correlation between DA ratio and aneurysm neck (R = 0.95, *P* = 0.0075), inverse correlation between LA ratio and aneurysm neck (R = 0.22, *P* = 0.0219) (Fig. 5).

In 115 patients with MCA bifurcation aneurysms, 94.8% (109 cases) aneurysms were deviated towards the side of the smaller lateral angle (φ2) (*P* < 0.001, Fig. 2B), 80.9% (93 cases) aneurysms were deviated towards the smaller daughter vessel (D2) (*P* < 0.001, Fig. 2C) and 77.4% (89 cases) aneurysms deviated towards both the smaller lateral angle and the smaller daughter vessel.

## Discussion

In recent years, it has been realized that morphologic and hemodynamic parameters play important roles in initiation and progression of cerebral aneurysms, and widened cerebral bifurcation angles and high vascular curvature, in particular, were associated with aneurysmal presence^7–9,13^. Alteration of bifurcation morphologic factors triggers changes in hemodynamic stresses^14^. Cerebral arterial tree follows the optimal work principle that establishes a balance between energy dissipation due to frictional resistance of blood flow and bifurcation geometry^15^, and arterial bifurcations harboring aneurysms usually disobey the optimal principle and experience abnormally increased hemodynamic stresses at the bifurcation apex^16^, suggesting that the morphology of arterial bifurcations is closely related to hemodynamic stresses and aneurysm formation. Our previous studies have demonstrated association of aneurysms at bifurcations of the anterior communicating and the basilar arteries with widened bifurcation angles and vascular diameters^8,9^. We thus analyzed the anatomical differences in patients with and without MCA bifurcation aneurysms and investigated possible morphological parameters related to MCA bifurcation aneurysm formation.

In patients with aneurysms at bifurcations of MCA, internal carotid artery and basilar artery, the bifurcation angle is greater than in patients with no bifurcation aneurysms^10,17,18^. In our study, we investigated the bifurcation angle in patients with or without MCA bifurcation aneurysms, and the MCA bifurcation angle between two M2 segments was shown to be significantly (*P* < 0.0001) greater in patients with than without MCA bifurcation aneurysms (152.4 ± 35.0° vs. 102.8 ± 24.7°). Furthermore, the lateral angles (φ2 and φ3) of aneurysmal bifurcation (76.6 ± 22.9° and 122.2 ± 24.1°, respectively) were significantly smaller than those of the control subjects without bifurcation aneurysms (111.3 ± 19.3° and 138.2 ± 14.8°, respectively) (*P* < 0.001) (Table 2 and Fig. 3), which was in agreement with previous reports^8,9,17^. This indicated that MCA bifurcation aneurysms are associated with wider MCA bifurcation angles and narrower lateral angles. Aneurysms formation at widened MCA bifurcations is probably related to abnormally enhanced hemodynamic stresses accompanied with widened bifurcations. It has been demonstrated that there is a densely packed band of collagen fibers at arterial bifurcation apex which can provide strength and stiffness to this area for protecting the bifurcation apex from abnormally enhanced hemodynamic stresses^1,19^. Computational fluid dynamic analysis showed that the direct flow impinging region on the arterial bifurcation apex is very small in narrowed bifurcations but very large in widened bifurcations^17,20,21^. The direct flow impinging region on the bifurcation apex may be protected by the densely packed fiber band in narrowed bifurcations, but in widened bifurcations, the direct flow impinging region could be well expanded beyond the protection band of fibers to produce abnormally increased hemodynamic stresses and subsequently cause destructive damage to the vascular wall leading to aneurysm initiation and formation. In clinical practice, the deployment of one or two stents at arterial bifurcations for the treatment of bifurcation aneurysms led to immediate and delayed bifurcation angle narrowing^22^, displaced and attenuated the flow impingement zone, and decreased the hemodynamic stresses at the bifurcation apex^20,21^ required for aneurysm recurrence or initiation.

Measurement of vascular diameter at the MCA bifurcations demonstrated that M1 (D1) and the smaller M2 segment (D2) were significantly (*P* = 0.006 and *P* < 0.001, respectively) smaller in patients with than without MCA bifurcation aneurysms (2.5 ± 0.8 mm and 1.6 ± 0.6 mm vs. 2.8 ± 1.0 mm and 1.9 ± 0.7 mm, respectively), whereas the DA and LA ratios were significantly (*P* < 0.0001) larger in patients with than without bifurcation aneurysms (1.6 ± 0.5 and 1.8 ± 0.7 vs. 1.4 ± 0.4 and 1.3 ± 0.3, respectively). In control subjects without MCA bifurcation aneurysms, smaller DA and LA ratios may indicate that two lateral angles or two M2 segments do not differ much in size, suggesting a more balanced or symmetrical MCA bifurcation in patients without MCA bifurcation aneurysms. However, in MCA bifurcations with aneurysms, larger DA and LA ratios may indicate that aneurysmal MCA bifurcation is more imbalanced or asymmetrical, with greater differences between two lateral angles or two M2 segments. This finding was in agreement with the results by Sadatomo *et al*.^2^, who showed that normal MCA bifurcations had close to a symmetric structure in the M2 segment and the lateral angles while aneurysmal MCA bifurcations did not have this symmetry. In their study^2^, the DA ratio was 1.5 ± 0.4 in normal MCA bifurcations but 1.7 ± 0.7 in aneurysmal MCA bifurcations, and the LA ratio was 1.3 ± 0.4 in normal patients but 2.2 ± 1.4 in aneurysmal MCA bifurcations, suggesting that MCA bifurcations with asymmetrical branches and asymmetrical lateral angles are more vulnerable to aneurysm formation at the MCA bifurcation.

The present study showed that LA ratio, DA ratio, bifurcation angles, D1 and D2 diameters were significantly (*P* < 0.05) different between MCA bifurcations with and without aneurysms, and we thus performed ROC analysis to evaluate aneurysm presence-associated morphological parameters. The results showed that angle φ1 was the best performer in discriminating between aneurysmal and nonaneurysmal MCA, with the cutoff point of 125.0° (AUC, 0.89), and sensitivity and specificity of 81.4% and85.4%, respectively. The AUC for the LA ratio and DA ratio were 0.77 and 0.66, respectively, with the LA ratio playing a more important role in predicting aneurysm formation at the MCA bifurcation. These results infer that widened MCA bifurcation angle and more asymmetrical lateral angles can be used to predict aneurysm formation at the MCA bifurcation through possible induction of abnormally enhanced hemodynamic stresses. We thus speculated that deployment of one stent via the parent artery M1 into the M2 branch forming a smaller lateral angle with M1 or deployment of two stents through the parent artery into two branches in Y configuration for stent-assisted coiling of MCA aneurysms can efficiently enlarge the smaller lateral angle, narrow the bifurcation angle φ1, and decrease the LA ratio. The MCA bifurcation can thus become more symmetrical for a better effect of treatment and prevention of recurrence. Gao *et al*. applied two stents deployed in Y configuration for treating basilar bifurcation aneurysms and one stent deployment through the parent artery to one branch for treating aneurysms at the bifurcations of MCA, basilar, anterior cerebral and internal carotid arteries^20–22^. Stent deployment can significantly increase the lateral angles but decrease the bifurcation angle, and with the bifurcation morphology changing towards symmetrical structures, the direct flow impinging center becomes smaller, the total pressure and peak wall shear stress at the bifurcation apex wall are both significantly decreased compared before stenting^20–22^.

Given the vital diagnostic role of the bifurcation angle φ1 in aneurysm presence, the relative dependency of angle φ1 was performed by using multivariate analysis. With increase of patient’ age, the bifurcation angle was significantly increased but the lateral angles were significantly decreased (*P* < 0.0001 and *P* = 0.0005, respectively). As discussed before, widening of bifurcation angles may produce abnormally enhanced hemodynamic stresses beyond the protection of a densely packed band of collagen fibers, consequently causing destructive remodeling of vessel wall and aneurysm formation. These results were in line with previous reports about the bifurcation angle at the anterior communicating artery and the basilar artery^8,9,17^. Further investigation of the morphological parameters at the MCA bifurcations revealed that the bifurcation angle was in an inverse correlation with the diameter of the M1 segment (D1, *P* = 0.042). The diameter of the M1 segment (D1) was significantly smaller in patients with than without MCA bifurcation aneurysms. This may suggest that the bifurcation angle is associated with bifurcation vessel diameters. With increase of patients’ age, the bifurcation angle will widen while the M1 segment will decrease in diameter, and older patients with greater MCA bifurcation angles and smaller M1 segment are more vulnerable to aneurysm formation at the MCA bifurcation. However, what hemodynamic stresses can be caused by greater bifurcation angles and smaller M1 segment in relation to aneurysm initiation at the bifurcation apex needs further investigation using computational fluid dynamics analysis so as to elucidate the possible underlying mechanism of aneurysm initiation at this site.

The multivariate analysis using the least squares linear regression showed a significant positive linear correlation in aneurysm size with M1, M2, M3 diameter or aneurysm neck (*P* = 0.004*, P* = 0.015, *P* < 0.0001 and *P* < 0.0001, respectively), which is in line with our previous findings about the anterior cerebral artery and basilar artery bifurcations^8,9^. A significant inverse correlation between LA ratio and aneurysm neck was also observed (*P* = 0.0219), however, no significant positive correlation between the bifurcation angle and aneurysm size or aneurysm neck was observed (*P* = 0.066 and *P* = 0.093, respectively). These results may indicate that the aneurysm size is associated with the diameter of the vessels at the MCA bifurcation and aneurysm neck, regardless of the bifurcation angle. Shojima *et al*.^23^ have shown that aneurysm size is a morphological factor associated with rupture of cerebral aneurysm, and the bifurcation vessel diameter is thus likely to be a morphological factor to predict aneurysm size and rupture in clinical setting. However, more work needs to be done for clarifying the exact mechanism.

The aneurysm neck is the site of aneurysm initiation, and aneurysm deviation is based on the location of aneurysm neck. Aneurysm deviation may indicate the aneurysm initiation site where abnormal hemodynamic stresses exist and trigger aneurysm formation. Our research showed that 94.8% MCA aneurysms were deviated towards the side of the smaller lateral angle (*P* < 0.001), implying that greater deflection of blood flow and stronger vortexes in the M2 branch forming a smaller angle with the M1 segment may induce abnormally increased hemodynamic stresses to initiate an aneurysm. Furthermore, 80.9% MCA aneurysms were deviated towards the smaller daughter vessel (*P* < 0.001), and 77.4% aneurysms deviated towards both the smaller lateral angle and smaller daughter vessel, inferring that the branch vessel with a smaller diameter and consequently thinner vessel wall is more vulnerable to aneurysms initiation under abnormally increased hemodynamic stresses compared with the relatively large contralateral branch artery.

In summary, MCA bifurcations in patients with aneurysms have asymmetrical bifurcation structures with widened bifurcation angles, narrowed lateral angles, imbalanced lateral angles and M2 segments, with the cutoff bifurcation angle of 125.0° and lateral angle ratio of 1.57 for predicting MCA bifurcation aneurysms, whereas normal MCA bifurcations show close to symmetric structures in the lateral angles and M2 branches.
